# Data on Lipocalin 2 and phosphatidylinositol 3-kinase signaling in a methionine- and choline-deficient model of non-alcoholic steatohepatitis

**DOI:** 10.1016/j.dib.2017.06.048

**Published:** 2017-07-01

**Authors:** Anastasia Asimakopoulou, Erawan Borkham-Kamphorst, Eddy Van de Leur, Ralf Weiskirchen

**Affiliations:** Institute of Molecular Pathobiochemistry, Experimental Gene Therapy and Clinical Chemistry, RWTH University Hospital Aachen, Aachen, Germany

**Keywords:** Lipocalin 2, Fat, MCD, NASH, PIP3, Oxidative stress, Mitochondria

## Abstract

The data presented in this brief report support the research article “*Altered mitochondrial and peroxisomal integrity in lipocalin-2-deficient mice with hepatic steatosis*” [1, doi: 10.1016/j.bbadis.2017.04.006]. We tested whether the absence of Lipocalin-2 (LCN2) could dysregulate the phosphatidylinositol 3-kinase/protein kinase B (PI3K-PKB) pathway and hepatic homeostasis in Non-Alcoholic-Steatohepatitis (NASH). The article highlights the role of LCN2 in hepatic homeostasis.

**Specifications Table**

**Table:** 

Subject area	*Biology*
More specific subject area	*Liver biology*
Type of data	*Figures (Immunofluorescence staining, Western blot analysis)*
How data was acquired	*Microscope (Nikon ECLIPSE 80i)* *Chemiluminescent detection (Boehringer Mannheim GmbH Lumi-Imager)*
Data format	*Raw*
Experimental factors	*Liver sections were derived from WT and Lcn2*^*-/-*^*mice*[2]*after feeding a MCD or standard chow diet for 6 weeks.* *Protein extracts were prepared from the same livers for Western blot analysis.* *Hepatocytes were isolated from WT and Lcn2*^*-/-*^*mice for in vitro experimentation.*
Experimental features	*Liver sections from WT and Lcn2*^*-/-*^*mice were immunostained for PIP3. Negative and positive controls were also used.* *100 µg of protein extracts from livers were used to detect PKB, phospho PKB, LCN2 and* Glyceraldehyde 3-phosphate dehydrogenase (*GAPDH) by Western blot.* *Hepatocytes were cultured from WT and Lcn2*^*-/-*^*mice. These were left untreated or treated with bovine insulin to induce PI3K and PIP3. Respective cells were immunostained for PIP3.*
Data source location	*Aachen, Germany*
Data accessibility	*Data not deposited elsewhere outside this article. Primary data are published in**[1]*.

**Value of the data**

• LCN2 is a versatile molecule participating in several pathways of hepatic homeostasis. The data describe the regulation of the PI3K/PIP3/PKB pathway with regard to the presence of LCN2 in a NASH model.
• The data study the value of LCN2 in PI3K signaling in NASH.
• The data are useful to understand how the absence of LCN2 affects PIP3 production.
• Signaling analyses could lead to novel treatment strategies to modulate medical conditions where the PI3K-PKB signaling pathway is dysregulated such as fatty liver and diabetes type 2 [3].
• The data presented in this article, could be compared with data from other animal NASH models to verify the strength of the effect. Moreover, comparison of this data to human NASH data on PI3K signaling and PIP3 functions could drive the development of novel LCN2 targeted therapies.

## 1 Data

**Fig. 1:**
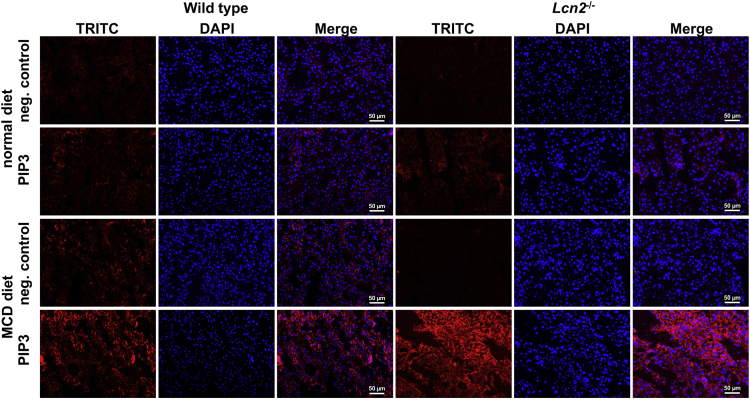
Immunodetection of phosphoinositide (3,4,5) triphosphate (PIP3) in liver cryosections. Immunostaining with a tetramethylrhodamine (TRITC)-conjugated antibody to detect PIP3 in mouse liver cryosections of each group (wc = wild type mouse standard diet; wm = wild type mouse MCD diet, kc = *Lcn2*^-/-^ mouse standard diet and km = *Lcn2*^-/-^ mouse MCD diet). Nuclei were counterstained with 4′,6-diamidino-2-phenylindole (DAPI). As a negative control, non-specific antiserum was used for staining. Scale bars, 50 µm.

**Fig. 2:**
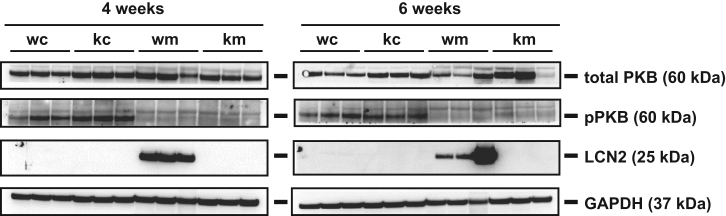
Visualization of expression of selected proteins by Western blot analysis. Protein extracts were prepared from livers of WT and *Lcn2*^-/-^ mice that were subjected to indicated diets for 4 or 6 weeks (*n*=3 animals/group). Antibodies used are mentioned in the Section 2. GAPDH was used as the loading control.

**Fig. 3:**
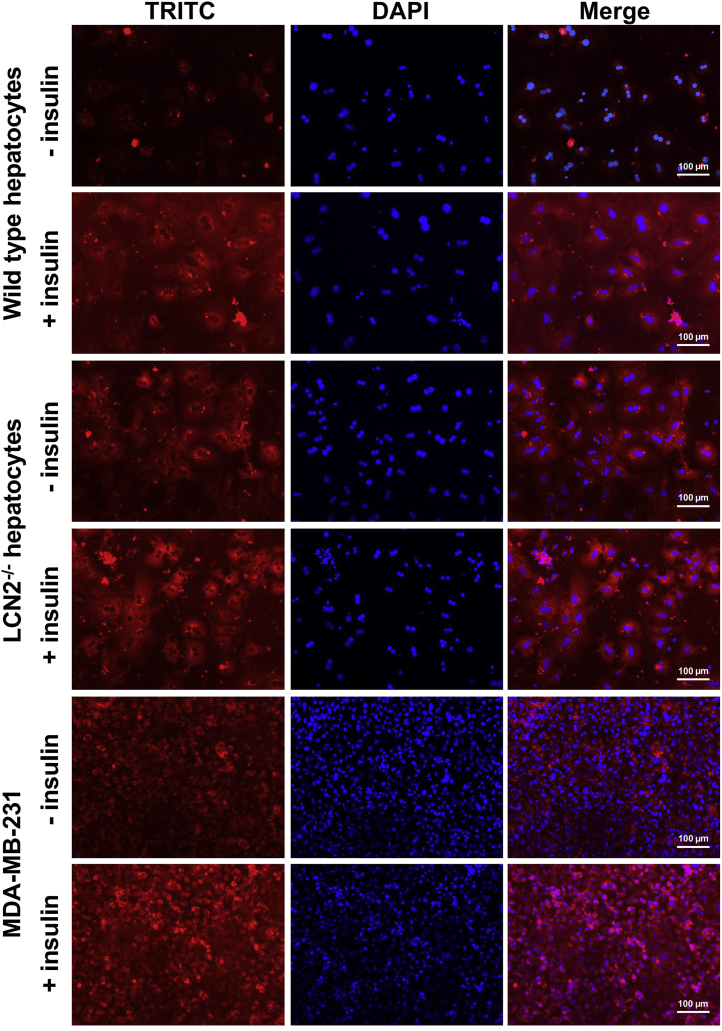
Immunodetection of PIP3 in cultured hepatocytes. Cultured hepatocytes isolated from WT or *Lcn2*-deficient animals fed normal chow were left untreated (−) or stimulated with 2 µg/ml bovine insulin (+) for 30 min. PIP3 in fixed cells was detected immunocytochemically using a tetramethylrhodamine (TRITC)-conjugated goat anti-mouse IgM specific for PIP3. Nuclei were counterstained with DAPI. The highly sensitive to insulin breast cancer cell line MDA-MB-231 [4] was used as a positive control. Immunostaining with a non-specific antiserum served as a negative control (data not shown). Scale bars, 100 µm.

The data include protein detection of members of the PI3K signaling and PIP3 quantification Fig. 1, Fig. 2, Fig. 3. The detection of respective biomolecules was done with fluorescence immunohistochemistry/immunocytochemistry and Western blot. The analyzed liver sections and primary hepatocytes originated from WT and *Lcn2*^-/-^ mice fed on a standard chow diet or an MCD diet. The hepatocytes were treated with insulin to trigger PIP3 and PI3K before immunodetection. Untreated control cells were used to compare.

## 2 Experimental design, materials and methods

### 2.1 Liver cryosections

Mice, WT and LCN2 deficient, were sacrificed under isoflurane (Forene®) anaesthesia (Abbott, Wiesbaden, Germany), and whole livers were resected. The livers were snap frozen in liquid nitrogen after they were covered with Tissue TEK OCT solution (Sakura Finetek Europe, Alphen aan den Rijn, The Netherlands) and kept at −80 °C. The snap frozen tissues were sliced into 5 μm standard sections and kept at −80 °C.

### 2.2 Cell culture and PIP3 stimulation

Primary hepatocytes from WT or *Lcn2*^-/-^ mice were isolated using the collagenase method of Seglen [5]. The hepatocytes were seeded on collagenase-coated cover slips in 6-well plates with HepatoZYME SFM (Thermo Fisher Scientific, Dreieich, Germany) supplemented with 2 mM L-glutamine (Sigma Aldrich, Taufkirchen, Germany) and 1% penicillin/streptomycin (Sigma Aldrich). They were cultivated for 2 days at 37 °C in incubator conditions of 5% CO_2_ and 100% humidity. Hepatocytes were left untreated or stimulated with bovine insulin (2 µg/ml) (Sigma Aldrich) for 30 min to trigger activation of PI3K and formation of PIP3 [6]. Stimulated hepatocytes were washed three times with ice cold PBS and fixed in 4% paraformaldehyde for 20 min at room temperature. As a control, MDA-MB-231 breast cancer cells [4], which are known to be highly sensitive to insulin [7], were cultivated in Dulbecco׳s Modified Eagle׳s medium (Sigma Aldrich) supplemented with 10% fetal bovine serum (Sigma Aldrich) prior to stimulation and fixation as described above.

### 2.3 Immunodetection of PIP3

PIP3 quantification in fixed cells and cryosections was performed using standard immunocytochemical methods [8]. Briefly, fixed cells and cryosections were washed three times with Tris-buffered saline (TBS), permeabilized in 0.5% saponin (Sigma Aldrich, cat. no. 47063-506-F) for 15 min at room temperature, and washed again three times with TBS. Cells were blocked with 10% (v/v) normal goat serum (DAKO, Hamburg, Germany, cat. no. X0907) in TBS for 1 h at 37 °C, followed by three washes in TBS containing 1% (v/v) normal goat serum. Samples were incubated overnight at 4 °C in TBS containing a 1:100 dilution of a mouse anti-PIP3 monoclonal antibody (Echelon Biosciences Inc., Salt Lake City, UT, USA, cat. no. Z-P345), 1% (w/v) bovine serum albumin (BSA) (Sigma Aldrich), and 1% (v/v) normal goat serum, respectively. This monoclonal antibody reacts primarily with the head group of PIP3 and has low cross-reactivity with other phosphoinositides [9]. Samples were washed three times in TBS containing 1% BSA and 1% normal goat serum and incubated for 1 h at RT with a TRITC-conjugated Affini Pure goat anti-mouse IgM specific for the µ chain (Jackson ImmunoResearch Laboratories Inc., Dianova, Hamburg, Germany, cat. no. 115-025-020) at a dilution of 1:200 in TBS supplemented with 1% BSA and 1% normal goat serum. After washes in TBS and distilled water, stained cells were mounted in mounting medium with DAPI (Vector Laboratories Inc., Burlingame, CA, cat. no. H-1200) .

### 2.4 Western blot

Equal amounts of total protein 100 μg/lane from liver tissue extracts were mixed with Nu-PAGE™ LDS electrophoresis sample buffer containing dithiothreitol as a reducing agent. Samples were heated at 87 °C for 10 min before fractionation on 4–12% Bis-Tris gels in 2-(N-morpholino) ethanesulfonic acid (MES) running buffer (Roth, Karlsruhe, Germany). Proteins were electroblotted onto a Protran membrane (GE Healthcare, Freiburg im Breisgau, Germany), and equal protein loading was confirmed by Ponceau S (Sigma) staining. For Western blotting, the membranes were first blocked by incubation in TBS supplemented with 1% Tween20 (TBST), containing 5% (w/v) non-fat milk powder (Roth). Primary antibodies used were: PKB (Cell Signaling Technology, Danvers, MA, USA, cat. no. 4685), p-PKB (Cell Signaling Technology, cat. no. 4060), LCN2 (R&D Systems, Minneapolis, MN, USA, cat. no AF3508), and GAPDH (Santa Cruz Biotech., Santa Cruz, CA, USA, cat. no sc-32233). The antibodies were diluted in 2.5% (w/v) non-fat milk powder or 2.5% (w/v) BSA in TBST prior to application to the membrane. Primary antibodies were visualized with anti-mouse-, anti-rabbit or anti-goat IgG secondary antibodies (all from Santa Cruz Biotech, CA cat. nos. sc-2004, sc-2005, sc-2056) in the Lumi-Imager (Roche Diagnostics, Darmstadt, Germany) using the SuperSignal chemiluminescent substrate (Thermo Fisher Scientific).

## Funding sources

This work was financially supported by Grants from the German Research Foundation (SFB TRR57, P13 and Q03) and the Interdisciplinary Centre for Clinical Research within the Faculty of Medicine at RWTH Aachen University (IZKF E6-11).

## References

[1] Asimakopoulou A., Fülöp A., Borkham-Kamphorst E., Van de Leur E., Gassler N., Berger T., Beine B., Meyer H.E., Mak W.T., Hopf C., Henkel C., Weiskirchen R. (1863 (2017) 2093-2110.). Altered mitochondrial and peroxisomal integrity in lipocalin-2-deficient mice with hepatic steatosis. Biochem. Biophys. Acta.

[2] Berger T., Togawa A., Duncan G.S., Elia A.J., You-Ten A., Wakeham A., Fong H.E., Cheung C.C., Mak T.W. (2006). Lipocalin 2-deficient mice exhibit increased sensitivity to Escherichia coli infection but not to ischemia-reperfusion injury. Proc. Natl. Acad. Sci. USA.

[3] Matsuda S., Kobayashi M., Kitagishi Y. (2013). Roles for PI3K/AKT/PTEN pathway in cell signaling of Nonalcoholic Fatty Liver Disease. ISRN Endocrinol..

[4] Cailleau R., Young R., Olivé M., Reeves W.J. (1974). Breast tumor cell lines from pleural effusions. J. Natl. Cancer Inst..

[5] Seglen P.O. (1976). Preparation of isolated rat liver cells. Methods Cell Biol..

[6] Band C.J., Mounier C., Posner B.I. (1999). Epidermal growth factor and insulin-induced deoxyribonucleic acid synthesis in primary rat hepatocytes is phosphatidylinositol 3-kinase dependent and dissociated from protooncogene induction. Endocrinology.

[7] Weichhaus M., Broom J., Wahle K., Bermano G. (2012). A novel role for insulin resistance in the connection between obesity and postmenopausal breast cancer. Int. J. Oncol..

[8] Niswender K.D., Gallis B., Blevins J.E., Corson M.A., Schwartz M.W., Baskin D.G. (2003). Immunocytochemical detection of phosphatidylinositol 3-kinase activation by insulin and leptin. J. Histochem. Cytochem..

[9] Chen R., Kang V.H., Chen J., Shope J.C., Torabinejad J., DeWald D.B., Prestwich G.D. (2002). A monoclonal antibody to visualize PtdIns(3,4,5)P_3_ in cells. J. Histochem. Cytochem..

